# Higher skeletal muscle mass associates with higher measured glomerular filtration rate in healthy individuals

**DOI:** 10.1093/ndt/gfae217

**Published:** 2024-10-03

**Authors:** Lisa B Westenberg, Marco van Londen, Marcel Zorgdrager, TransplantLines Investigators, Martin H de Borst, Alain R Viddeleer, Stephan J L Bakker, Robert A Pol

**Affiliations:** Department of Surgery, Division of Transplant Surgery; Department of Internal Medicine, Division of Nephrology; Department of Internal Medicine, Division of Nephrology; Department of Radiology, Medical Imaging Center, University Medical Center Groningen, University of Groningen, Groningen, the Netherlands; Department of Surgery, Division of Transplant Surgery; Department of Internal Medicine, Division of Nephrology; Department of Internal Medicine, Division of Nephrology; Department of Radiology, Medical Imaging Center, University Medical Center Groningen, University of Groningen, Groningen, the Netherlands; Department of Internal Medicine, Division of Nephrology; Department of Surgery, Division of Transplant Surgery

To the Editor,

Low muscle mass is a prevalent issue among patients with chronic kidney disease [1] and has been associated with adverse outcomes, including increased risk of mortality and end-stage kidney disease [2]. Improving muscle mass and function shows beneficial effects on kidney function [3–6], suggesting that muscle mass and performance are associated with kidney function. Many aspects of this association remain unexplored. This study aimed to investigate whether an association between skeletal muscle and measured glomerular filtration rate (mGFR, ^125^I-Iothalamate) exists in healthy individuals, investigating computed tomography (CT) -derived muscle mass measurements, muscle quality, and 24-hour urinary creatinine excretion rate (24-h CER). We hypothesized that higher muscle mass, quality, and 24-h CER levels are associated with higher mGFR.

In this cross-sectional study, skeletal muscle area (SMA) and density (skeletal muscle radiation attenuation, SMRA) were based on CT-scans at vertebral level L3. Muscle area was indexed for height (skeletal muscle index, SMI), and SMA, SMI, and SMRA were stratified by age. The 24-h CER was assessed as a biochemical marker for muscle mass. Participants underwent GFR measurements. An extensive description of the methodology is provided in Annex I of the Online Supplementary Material.

In total, 888 healthy participants were included, 49% of whom were male (Table 1). Age was 53 ± 11 years, body mass index (BMI) was 26.2 ± 3.41 kg/m^2^, and body surface area (BSA) was 1.96 ± 0.19 m^2^. The 24-h CER was 13.6 ± 4.64 mmol/24 hours, SMA was 147.8 ± 34.6 cm^2^, SMI was 47.6 ± 8.38 cm^2^/m^2^, and SMRA was 48.6 ± 7.52 Hounsfield units (HU). Non-standardized mGFR was 112.5 ± 21.1 ml/min, and BSA-standardized mGFR was 99.3 ± 15.5 ml/min per 1.73 m^2^.

**Table 1:** Baseline characteristics of the study population.

Variable	All participants (*N* = 888)	≤53 years (*n* = 433)	>53 years (*n* = 455)
Sex			
Female, *n* (%)	453 (51)	219 (51)	234 (51)
Male, *n* (%)	435 (49)	214 (49)	221 (49)
Age at CT, years	53 ± 11	44 ± 7	62 ± 6
Weight, kg	80.5 ± 13.1	81.4 ± 13.3	79.7 ± 12.8
Height, cm	175.3 ± 9.47	176.6 ± 9.55	174.1 ± 9.24
BMI, kg/m^2^	26.2 ± 3.41	26.1 ± 3.49	26.2 ± 3.35
BSA, m^2^	1.96 ± 0.19	1.98 ± 0.19	1.94 ± 0.19
Systolic blood pressure, mmHg	126.5 ± 12.9	123.4 ± 11.1	129.5 ± 13.8
**Muscle parameters**			
SMA, cm^2^	147.8 ± 34.6	153.2 ± 34.9	142.6 ± 33.7
Skeletal muscle index, cm^2^/m^2^	47.6 ± 8.38	48.7 ± 8.33	46.6 ± 8.31
Skeletal muscle radiation attenuation, HU	48.6 ± 7.52	51.0 ± 6.71	46.2 ± 7.51
Urinary creatinine excretion rate, mmol/24 hours	13.6 ± 4.64	14.7 ± 4.71	12.6 ± 4.36
Height-indexed urinary creatinine excretion rate, mmol/24 hours/m	7.72 ± 2.44	8.27 ± 2.48	7.20 ± 2.29
Handgrip strength, *N* Dominant hand Non-dominant hand	41.5 ± 12.0 40.6 ± 12.6	43.3 ± 12.2 40.7 ± 13.2	40.0 ± 11.7 40.5 ± 12.1
**Renal function parameters**			
estimated glomerular filtration rate, ml/min/1.73 m^2^	86.8 ± 15.0	92.4 ± 15.0	81.7 ± 13.0
mGFR, ml/min	112.5 ± 21.1	120.9 ± 20.0	104.6 ± 19.0
BSA-standardized mGFR, ml/min per 1.73 m^2^	99.3 ± 15.5	105.7 ± 14.3	93.2 ± 14.1

A significant interaction was found for age with SMA, SMI, and SMRA. Therefore, the analyses were stratified for age (≤ or >mean age). SMA showed a statistically significant positive correlation with 24-h CER (*r* value = 0.66, *P* < .001). In univariable linear regression analyses, SMA, SMI, SMRA, and 24-h CER were significantly associated with non-standardized mGFR, with higher levels being associated with higher mGFR (Supplemental Table S1). Other determinants of mGFR in the total population were female sex (*Β* = −3.63, *P* < .001), systolic blood pressure (*Β* = −0.11, *P* = .01), and age (*Β* = −0.65, *P* < .001). After stratifying for age, systolic blood pressure was no longer significantly associated with mGFR.

After adjusting for sex, SMA, SMI, and SMRA, 24-h CER remained significantly associated with mGFR (SMA: younger individuals: *B* = 0.31, *P* < .001; older individuals: *B* = 0.33, *P* < .001; SMI: younger individuals: *B* = 0.57, *P* < .001; older individuals: *B* = 0.64, *P* < .001; SMRA: younger individuals: *B* = −0.35, *P* = .01; older individuals: *B* = −0.13, *P* = .23; 24-h CER: younger individuals: *B* = 1.12, *P* < .001; older individuals: *B* = 1.09, *P* < .001) (Table 2). In analyses adjusted for sex, BSA, and age, higher SMA and SMI were associated with higher mGFR (Table 2). SMRA was no longer significantly associated with mGFR in these analyses. The 24-h CER remained significantly associated with mGFR in those aged ≤53 years (*B* = 0.51, *P* = .02), but not in older individuals (*B* = 0.41, *P* = .06). When splitting the analyses with 24-h CER by sex, higher 24-h CER was associated with higher mGFR in younger female donors and older male donors (Supplemental Table S2).

**Table 2:** Multivariable linear regression analyses with mGFR, per age category.

	Age ≤53 years				Age >53 years			
mGFR (ml/min)	*B* (95% CI)	Std β	*P*	Adj. *R*^2^	*B* (95% CI)	Std. *β*	*P*	Adj. *R*^2^
SMA	0.31 (0.23; 0.39)	.55	<.001	.28	0.33 (0.25; 0.41)	.58	<.001	.34
Female sex	0.63 (−4.81; 6.07)	.02	.82		−0.22 (−5.48; 5.05)	−.01	.94	
SMI	0.57 (0.31; 0.84)	.24	<.001	.21	0.64 (0.38; 0.89)	.28	<.001	.28
Female sex	−10.8 (−15.2; −6.35)	−.27	<.001		−11.3 (−15.5; −7.10)	−.30	<.001	
SMRA	−0.35 (−0.60; −0.10)	−.12	.01	.19	−0.13 (−0.33; 0.08)	−.05	.23	.24
Female sex	−17.6 (−21.0; −14.2)	−.44	<.001		−19.1 (−22.2; −16.0)	−.50	<.001	
24-h CER	1.12 (0.68; 1.55)	.26	<.001	.23	1.09 (0.66; 1.52)	.25	<.001	.29
Female sex	−11.3 (−15.5; −7.24)	−.28	<.001		−13.3 (−17.0; −9.55)	−.35	<.001	
SMA	0.16 (0.07; 0.25)	.28	<.001	.35	0.24 (0.15; 0.33)	.42	<.001	.37
Female sex	1.61 (−3.59; 6.80)	.04	.54		0.34 (−4.84; 5.52)	.01	.90	
BSA	39.9 (28.1; 51.6)	.39	<.001		23.2 (12.2; 34.1)	.23	<.001	
SMI	0.43 (0.19; 0.68)	.18	<.001	.35	0.59 (0.35; 0.83)	.26	<.001	.36
Female sex	−0.55 (−5.08; 3.98)	−.01	.81		−2.97 (−7.53; 1.59)	−.08	.20	
BSA	48.8 (38.7; 58.9)	.47	<.001		36.4 (26.7; 46.1)	.36	<.001	
SMRA	0.23 (−0.04; 0.49)	.08	.09	.33	0.34 (0.11; 0.56)	.13	.003	.34
Female sex	−3.26 (−7.58; 1.05)	−.08	.14		−6.66 (−10.9; −2.46)	−.18	.002	
BSA	55.5 (43.9; 67.1)	.54	<.001		46.4 (35.0; 57.8)	.47	<.001	
24-h CER	0.49 (0.06; 0.92)	.12	.03	.34	0.57 (0.13; 1.02)	.13	.01	.35
Female sex	−3.27 (−7.51; 0.97)	−.08	.13		−7.98 (−12.0; −4.00)	−.21	<.001	
BSA	47.6 (36.4; 58.7)	.46	<.001		32.5 (21.9; 43.1)	.33	<.001	
SMA	0.15 (0.06; 0.23)	.25	<.001	.37	0.19 (0.10; 0.28)	.34	<.001	.41
Female sex	2.22 (−2.91; 7.35)	.06	.40		−2.69 (−7.76; 2.38)	−.07	.30	
BSA	40.6 (29.1; 52.2)	.39	<.001		21.4 (10.8; 31.9)	.21	<.001	
Age	−0.40 (−0.61; −0.19)	−.15	<.001		−0.77 (−1.01; −0.52)	.23	<.001	
SMI	0.43 (0.19; 0.67)	.18	<.001	.37	0.49 (0.26; 0.72)	.21	<.001	.41
Female sex	0.65 (−3.84; 5.15)	.02	.78		−5.12 (−9.53; −0.70)	−.14	.02	
BSA	48.6 (38.6; 58.5)	.47	<.001		31.6 (22.3; 41.0)	.32	<.001	
Age	−0.43 (−0.64; −0.21)	−.16	<.001		−0.80 (−1.04; −0.55)	−.24	<.001	
SMRA	0.07 (−0.21; 0.34)	.02	.63	.35	0.10 (−0.13; 0.33)	.04	.39	.39
Female sex	−3.08 (−7.34; 1.19)	−.08	.16		−9.71 (−13.9; −5.57)	−.26	<.001	
BSA	52.0 (40.4; 63.7)	.50	<.001		35.1 (23.6; 46.6)	.35	<.001	
Age	−0.40 (−0.63; −0.18)	−.15	<.001		−0.83 (−1.09; −0.57)	−.25	<.001	
24-h CER	0.51 (0.09; 0.93)	.12	.02	.36	0.41 (−0.02; 0.83)	.09	.06	.41
Female sex	−2.02 (−6.23; 2.18)	−.05	.35		−9.59 (−13.4; −5.75)	−.26	<.001	
BSA	47.1 (36.2; 58.1)	.45	<.001		28.8 (18.6; 38.9)	.29	<.001	
Age	−0.45 (−0.67; −0.23)	−.16	<.001		−0.81 (−1.06; −0.57)	−.25	<.001	

In secondary analyses with BSA-standardized mGFR, higher SMI, and also in older individuals, SMRA, was significantly associated with higher mGFR (Supplemental Table S3). The 24-h CER was not significantly associated with BSA-standardized mGFR.

Multivariable linear regression analyses with mGFR as an independent variable and the skeletal muscle parameters as dependent variables were performed as supplementary analyses. These analyses showed that mGFR was significantly associated with all skeletal muscle parameters except SMRA in analyses adjusted for sex, systolic blood pressure, BSA, and age (Supplemental Tables S4 and S5).

This study showed a significant association of higher CT-measured skeletal muscle mass with higher kidney function, and vice versa, in healthy individuals. To the best of our knowledge, this study is the first to show a significant association of CT-measured skeletal muscle mass with mGFR. A previous study involving 67 healthy young men with varying body mass, in which lean mass was determined using dual-energy X-ray absorptiometry, also highlighted the significance of lean mass as a determinant of GFR clearance by ^99^ ^m^Tc-DTPA [7]. Muscular men exhibited significantly higher GFR levels than those with normal BMI and obese participants [7]. Among patients with chronic kidney disease stages 3–5, loss of lean body mass was significantly related to decline in GFR [8]. For an extensive discussion, we refer to Annex II of the Online Supplementary Material.

Owing to the cross-sectional design of the study, it is not possible to derive causal relationships from the analyses. In fact, this study also found significant associations between mGFR and muscle quantity (CT-measured as well as through 24-h CER), and it is established in literature that low muscle mass is prevalent among patients with kidney disease [1, 9]. These studies postulate that low muscle mass in patients with chronic kidney disease is due to several causes often present in these patients, leading to protein imbalance with increased protein degradation and reduced protein synthesis [9]. Future studies are necessary to uncover causal pathways underlying muscle mass, muscle quality, and kidney function.

Certain study limitations are worth noting. Owing to the cross-sectional study design, it is not possible to derive causal relationships from the presented results. The use of contrast may have affected the results, particularly the skeletal muscle density measurements [10]. This study included almost solely individuals of European descent and, due to our center's inclusion criteria for living kidney donation, a limited number of individuals (*n* = 6) with a body size at the extreme end of the BMI spectrum (BMI >35 kg/m^2^), limiting generalizability to more ethnically diverse and obese populations.

This study shows that higher skeletal muscle mass is significantly associated with higher kidney function in healthy individuals. These findings call for further investigation of the underlying mechanisms and investigation of possible benefits of translation to clinical practice.

## Supplementary Material

gfae217_Supplemental_File
