# Supplementary material for: Higher skeletal muscle mass associates with higher measured glomerular filtration rate in healthy individuals
Source: Nephrol Dial Transplant. 2024 Oct 3;40(1):212–4. doi: 10.1093/ndt/gfae217 (PMC11659972; doi:10.1093/ndt/gfae217)
Supplement: gfae217_Supplemental_File [file gfae217_supplemental_file.docx]

**Online Supplementary Material**

**Table of contents**

Annex I: Extensive methodology Pages 2 – 4

Annex II: Extensive discussion Pages 5 – 9

Annex III: Supplementary Tables Pages 10 – 15

Annex IV: Supplementary Figures Pages 16 – 17

**Annex I: Extensive methodology**

**Materials and Methods**

*Subjects*

A total of 888 healthy subjects (potential living kidney donors) were included in this retrospective cross-sectional study at the University Medical Center Groningen (UMCG, Groningen, the Netherlands) between 2002 and 2019. All subjects were retrospectively reviewed for eligibility. Exclusion criteria were inability to provide informed consent, presence of significant interfering artefacts on CT imaging and/or in case the abdominal wall muscles were not fully visualized on the CT scan. For this study, all clinical and radiological data were retrieved from the TransplantLines Biobank and Cohort Study (ClinicalTrials.gov identifier: NCT03272841). All participants were ≥18 years of age and gave written informed consent on enrolment. A detailed description of the study design, inclusion and exclusion criteria has been published previously [1]. The study protocol has been approved by the local Institutional Review Board (METc 2014/077), adheres to the UMCG Biobank Regulation, and is in accordance with the WMA Declaration of Helsinki and the Declaration of Istanbul [1]. An overview of the study participants is displayed in Supplemental Figure S1.

*Data collection*

All subjects underwent CT imaging at the UMCG (n=882) or a non-academic referring hospital (n=6) in the Netherlands. The majority of CT scans were contrast-enhanced (n=885) (n=7 portal venous phase, n=31 arterial phase, n=847 late phase) and three scans were unenhanced. Slice thickness varied from 0.75 to 5 mm, but the majority (n=834, 94%) had a thickness of 2 mm. Tube voltage and current varied from 70-150 kVp (median 100 [100-100] kVp) and 1-455 mAs (mean 97.8 ± 47.1 mAs), respectively. The skeletal muscle area (SMA, cm^2^) was determined as described previously [2] and included psoas, paraspinal and abdominal wall muscles at vertebral level L3. SMA was indexed for height of the subject (skeletal muscle index, SMI, cm²/m²). Muscle tissue was defined by selecting voxels with densities ranging from -29 to +150 Hounsfield units (HU). Muscle quality was quantified by examining muscle density (i.e., skeletal muscle radiation attenuation, SMRA), and defined as mean HU of all pixels of the total SMA. Kidney function was quantified as mGFR using the ^125^I-Iothalamate infusion method, which was detailed in a previous publication by our group [3]. All clinical and biochemical parameters, among which 24-h CER, were collected within a time frame of one week before or after cross-sectional imaging and were conducted following the protocols outlined in a previous publication [1].

*Statistical analysis*

Normally distributed continuous variables were presented as mean (standard deviation). A correlation analysis was performed with 24-h CER and SMA. Glomerular filtration rate is known to be influenced by age [4], sex [5], and body size [6]. Uni- and multivariable linear regression analyses were performed to assess possible associations with mGFR. Primary analyses were performed with mGFR that was not standardized for BSA. Secondary analyses were performed with mGFR that was standardized for measured body surface area following the DuBois formula (BSA, m^2^), yielding mGFR in ml/min per 1.73 m^2^, a common practice in literature. No significant deviation from linearity was found for SMI, SMRA, 24-h CER, and BSA in the study population with all participants. Possible interaction effects for age, sex, and BMI with the skeletal muscle parameters were analyzed. The definitive model for the multivariable linear regression analyses contained: SMA (cm^2^) or SMI (cm²/m²) or SMRA (HU) or 24-h CER (mmol/24 hours), sex, BSA (m^2^, in primary analyses). 24-h CER was indexed for height (m) in additional analyses. The effect of not only stratifying for age, but also adding age as a covariable was examined as well. In light of the possibility of reverse causality in a cross-sectional study, multivariable linear regression analyses with mGFR as an independent variable and the skeletal muscle parameters as dependent variables were performed as supplementary analyses. Missing data was limited (≤5%). Participants who did not have muscle quality or 24-h CER data were not included in the regression analyses for which they had missing muscle data. Homoscedasticity and normal distribution of residuals were present for all models. Durbin-Watson test was used to check autocorrelation of residuals. Variance inflation factor diagnostics showed that no collinearity was present. Two-tailed values were used, and significance level was set at p-value below 0.05. The analyses were performed using SPSS® version 28 (IBM, Armonk, NY, USA) and RStudio (PBC, Boston, MA, USA, 2021).

References:

1. Eisenga MF, Gomes-Neto AW, van Londen M, Ziengs AL, Douwes RM, Stam SP, et al. Rationale and design of TransplantLines: a prospective cohort study and biobank of solid organ transplant recipients. *BMJ Open*. 2018;8(12):e024502.
2. Westenberg LB, Zorgdrager M, Swaab TDA, van Londen M, Bakker SJL, Leuvenink HGD, et al. Reference values for low muscle mass and myosteatosis using tomographic muscle measurements in living kidney donors. *Sci Rep*. 2023;13(1):5835.
3. van Londen M, Kasper N, Hessels NR, Messchendorp AL, Bakker SJL, Sanders JS, et al. Renal functional reserve capacity before and after living kidney donation. *Am J Physiol Renal Physiol*. 2018;315(6):F1550-F4.
4. Denic A, Glassock RJ, Rule AD. Structural and Functional Changes With the Aging Kidney. *Adv Chronic Kidney Dis*. 2016;23(1):19-28.
5. Berg UB. Differences in decline in GFR with age between males and females. Reference data on clearances of inulin and PAH in potential kidney donors. *Nephrol Dial Transplant*. 2006;21(9):2577-82.
6. Geddes CC, Woo YM, Brady S. Glomerular filtration rate--what is the rationale and justification of normalizing GFR for body surface area? *Nephrol Dial Transplant*. 2008;23(1):4-6.

**Annex II: Extensive discussion**

We hypothesized that higher muscle mass, quality, and 24-h CER levels are associated with higher mGFR. This study showed a significant association of higher CT-measured skeletal muscle mass with higher kidney function, and vice versa, in healthy individuals.

Assessing the relationship between muscle mass and kidney function has proven challenging due to the direct influence of muscle mass on estimates of kidney function, especially when using creatinine-based calculations of glomerular filtration rate. In clinical practice, kidney function is frequently assessed by estimated glomerular filtration rate (eGFR). While eGFR equations do take into account certain factors such as sex and age [1, 2], recent research has shown a tendency to overestimate eGFR in individuals with low muscle mass and muscle wasting [3]. In addition to eGFR equations incorporating Cystatin C, which is much less dependent on muscle mass than creatinine, efforts have been made to develop and validate creatinine-based eGFR equations that incorporate CT analysis of muscle mass [4]. The method considered most precise for measuring GFR is however through the calculation of the clearance of an exogenous filtration marker, such as ^125^I-Iothalamate, which is known as measured GFR (mGFR) [5]. This approach is unaffected by variation in muscle mass, which allows for a thorough examination of the relationship between muscle mass and actual kidney function. Advances in CT-analysis have provided a more direct means of measuring both muscle mass and muscle density, with the latter serving as a marker for muscle quality, as compared to the commonly used biochemical marker of muscle mass 24-hour urinary creatinine excretion rate (24-h CER). While only two studies have employed radiological muscle mass assessments to investigate a possible association between muscle mass and kidney function, these studies have pointed to lean mass as a determinant of mGFR in patients with chronic kidney disease [6] and men of normal and extreme body composition [7].

To the best of our knowledge, this study is the first to show a significant association of CT-measured skeletal muscle mass with kidney function, expressed as mGFR. A prior study involving 67 healthy young men with varying body mass, in which lean mass was determined using dual-energy X-ray absorptiometry, also highlighted the significance of lean mass as a determinant of GFR clearance by ^99m^ Tc-DTPA [7]. Notably, muscular men exhibited significantly higher GFR levels than those with normal BMI and obese participants [7]. Among patients with chronic kidney disease stages 3-5, loss of lean body mass was significantly related to decline in GFR [6].

Expanding upon these prior findings, the secondary analyses with BSA-standardized mGFR in this study additionally show that higher muscle quality (denoted by higher skeletal muscle radiation attenuation, or muscle density) was significantly associated with higher kidney function. Decreased skeletal muscle radiation attenuation or muscle density on CT analysis is indicative of excessive fat deposition in muscle, a condition known as myosteatosis. Myosteatosis has garnered attention for its detrimental effects on clinical outcomes across various patient groups. In patients with kidney failure, myosteatosis has previously been associated with higher incidence of cardiovascular disease [8] and increased mortality [9]. Keddar et al. showed that in patients undergoing peritoneal dialysis, as kidney function declined, there was a progressive accumulation of fat in skeletal muscle [8]. Moreover, when compared to subjects with normal kidney function, those with kidney failure generally exhibited lower muscle radiation attenuation [9]. In younger participants, after adjusting for age (in addition to sex), the association between muscle quality (SMRA) and mGFR was no longer statistically significant. In these younger individuals, age was a stronger determinant of mGFR, with higher age being associated with lower mGFR. It may be possible that the relationship of muscle quality and mGFR strengthens with older age, as the association between muscle quality and mGFR remained significant after adjusting for both sex and age in older individuals.

Due to the cross-sectional design of the study, it is not possible to derive causal relationships from the analyses. In fact, this study also found significant associations between mGFR and muscle quantity (CT-measured as well as through 24-h CER) and it is established in literature that low muscle mass is prevalent among patients with kidney disease [10, 11]. These studies postulate that the existence of low muscle mass in patients with chronic kidney disease is due to a number of causes that are often present in these patients, leading to a protein imbalance with increased protein degradation and reduced protein synthesis [11]. The varied perspectives within the literature regarding the relationship between muscle and kidney function, as well as regarding the direction of causation, invite further exploration in future studies to uncover causal pathways underlying muscle mass, muscle quality, and kidney function.

The measurement of 24-h CER is widely utilized to evaluate muscle mass [12], with higher values generally indicative of greater muscle mass. In primary analyses, 24-h CER was associated with non-standardized mGFR levels when adjusting for sex, BSA, and age. In secondary analyses, both crude and height-indexed 24-h CER were found to be significantly associated solely with BSA-standardized mGFR levels in univariable linear regression analyses and lost significance after adjusting for sex. This suggests that 24-h CER may primarily reflect body size or BSA, rather than muscle mass. BSA standardization is commonly used in literature to account for the influence of body weight and height on kidney function and may allow for a more specific assessment of the association between muscle mass and kidney function in these individuals, relatively independent of their overall body size. In this study, both BSA-standardized and non-standardized mGFR were analyzed. In addition, an important difference between CT-measured skeletal muscle mass and 24-h CER is that 24-h CER is a marker of muscle mass that has been degraded and CT analysis of muscle mass measures the actual volume of skeletal muscle. Therefore, the rate of metabolic turnover of muscle becomes a factor of influence when investigating 24-h CER as an indicator of muscle mass, a factor that could not be accounted for in the present study.

*Underlying mechanisms*

When examining the relationship between muscle and kidney function, one encounters the question of causality. Most studies have focused on the existence of a relationship between kidney function and muscle in patients with chronic kidney disease, where a decline in kidney function is associated with a reduction in muscle mass and strength [11]. The mechanisms underlying the relationship between kidney function and muscle are mostly attributed to an ongoing imbalance characterized by increased protein degradation, decreased protein synthesis in muscle tissue and decreased muscle regeneration [11, 13]. In patients with chronic kidney disease, this imbalance arises from processes such as inflammation, hormonal alterations, insulin resistance, metabolic acidosis, the hemodialysis process, and reduced endogenous creatine production [13, 14], as illustrated in Supplemental Figure S2. Few studies have delved into the opposite relationship: muscle mass having an effect on kidney function, and many of these studies were cross-sectional in nature, making them susceptible to the potential impact of reverse causality [6]. A longitudinal study with 8 years follow-up in patients with type 2 diabetes showed that every standard deviation above baseline skeletal muscle index (measured using bio-impedance analysis) was associated with an 18% lower risk of chronic kidney disease progression and a 17% lower risk of albuminuria progression [15]. They propose a role for biomarkers such as pigment epithelium-derived factor (PEDF), which has antiangiogenic, antioxidant, and anti-inflammatory effects [15]. The study by Kim et al. highlights the significance of insulin resistance in this context. In their study, they found that individuals with sarcopenia had an increased risk of albuminuria, and vice versa [16]. Skeletal muscle plays a pivotal role in insulin-mediated glucose disposal, and so a loss of muscle may potentially lead to insulin resistance and, ultimately, albuminuria [16].

In a large study performed by Cho et al., they found that higher BSA-adjusted total kidney volume was significantly associated with greater muscle volume, as well as higher eGFR, physical performance, and reduced risk of CKD [17]. Tanaka et al. observed a significant association between BSA-adjusted kidney parenchymal volume and a higher height-normalized SMI in non-diabetic participants [18]. They point at crosstalk between kidney and muscle as the mechanism underlying this relationship between kidney and muscle volume: myokines produced by skeletal muscle are linked to growth and regeneration of damaged kidney and the suppression of fibrogenesis in the kidney [19-23]. Kidney injury may in turn lead to the release of factors that affect skeletal muscle by reducing muscle homeostasis, leading to muscle wasting [24-28]. It could also be possible that individuals with higher muscle mass have a higher metabolic rate, which itself sets the GFR [29]. Much about the relationship between muscle and kidney function in healthy individuals with normal kidney function remains unknown. The physiological processes and causal pathways underlying this phenomenon deserve more attention in future research.

*Clinical interpretation*

This study provides valuable insights into the relationship between body composition and kidney function in healthy individuals. Skeletal muscle mass measurements and possible improvement of skeletal muscle quantity and quality might be of interest in clinical care for those at risk of kidney failure, kidney disease patients, as well as healthy individuals. Future studies should focus on the physiological mechanisms and causal pathways underlying the relationship between muscle and kidney function, with an aim to investigate possible benefits of translating this to clinical practice.

*Strengths and limitations*

This study has several notable strengths, such as its substantial cohort of healthy individuals, the use of standardized kidney function measurements, and the use of an objective measure of determining muscle characteristics (mass and quality) through CT scan analysis. In addition, this study not only uses muscle mass measurements to investigate the association between skeletal muscle and kidney function, but also muscle quality. However, certain limitations are worth noting. Due to the cross-sectional study design, it is not possible to derive causal relationships from the presented results. The use of contrast may have impacted the results, particularly the skeletal muscle density measurements [30]. This study included almost solely individuals of European descent, and, due to our center’s inclusion criteria for living kidney donation, a limited number of individuals (n=6) with a body size at the extreme end of the BMI-spectrum (BMI >35 kg/m^2^), limiting generalizability to more ethnically diverse and obese populations. And lastly, there are no data on interobserver variability and day-to-day variability of SMA and SMRA measurements. It would be of interest to acquire such data in a future study.

*Conclusion*

This study shows that higher skeletal muscle mass is significantly associated with higher kidney function in healthy individuals. In older individuals, higher skeletal muscle quality was also associated with higher kidney function. These findings call for further investigation of the underlying mechanisms and investigation of possible benefits of translation to clinical practice.

References:

1. Levey AS, Stevens LA, Schmid CH, Zhang YL, Castro AF, 3rd, Feldman HI, et al. A new equation to estimate glomerular filtration rate. *Ann Intern Med*. 2009;150(9):604-12.
2. Inker LA, Schmid CH, Tighiouart H, Eckfeldt JH, Feldman HI, Greene T, et al. Estimating glomerular filtration rate from serum creatinine and cystatin C. *N Engl J Med*. 2012;367(1):20-9.
3. Groothof D, Post A, Polinder-Bos HA, Erler NS, Flores-Guerrero JL, Kootstra-Ros JE, et al. Muscle mass and estimates of renal function: a longitudinal cohort study. *J Cachexia Sarcopenia Muscle*. 2022;13(4):2031-43.
4. Stehlé T, Ouamri Y, Morel A, Vidal-Petiot E, Fellahi S, Segaux L, et al. Development and validation of a new equation based on plasma creatinine and muscle mass assessed by CT scan to estimate glomerular filtration rate: a cross-sectional study. *Clin Kidney J.* 2023;16(8):1265-1277.
5. Boele-Schutte E, Gansevoort RT. Measured GFR: not a gold, but a gold-plated standard. *Nephrol Dial Transplant*. 2017;32(suppl_2):ii180-ii4.
6. Zhou Y, Hellberg M, Svensson P, Hoglund P, Clyne N. Sarcopenia and relationships between muscle mass, measured glomerular filtration rate and physical function in patients with chronic kidney disease stages 3-5. *Nephrol Dial Transplant*. 2018;33(2):342-8.
7. Chew-Harris JS, Florkowski CM, Elmslie JL, Livesey J, Endre ZH, George PM. Lean mass modulates glomerular filtration rate in males of normal and extreme body composition. *Intern Med J*. 2014;44(8):749-56.
8. Keddar M, Muylle T, Carrie E, Trefois P, Nachit M, Crott R, et al. Non-invasive Quantification of Fat Deposits in Skeletal Muscle Predicts Cardiovascular Outcome in Kidney Failure. *Front Physiol*. 2020;11:130.
9. Morel A, Ouamri Y, Canoui-Poitrine F, Mule S, Champy CM, Ingels A, et al. Myosteatosis as an independent risk factor for mortality after kidney allograft transplantation: a retrospective cohort study. *J Cachexia Sarcopenia Muscle*. 2022;13(1):386-96.
10. Sabatino A, Cuppari L, Stenvinkel P, Lindholm B, Avesani CM. Sarcopenia in chronic kidney disease: what have we learned so far? *J Nephrol*. 2021;34(4):1347-72.
11. Stenvinkel P, Carrero JJ, von Walden F, Ikizler TA, Nader GA. Muscle wasting in end-stage renal disease promulgates premature death: established, emerging and potential novel treatment strategies. *Nephrol Dial Transplant*. 2016;31(7):1070-7.
12. Forbes GB, Bruining GJ. Urinary creatinine excretion and lean body mass. *Am J Clin Nutr*. 1976;29(12):1359-66.
13. Cheng TC, Huang SH, Kao CL, Hsu PC. Muscle Wasting in Chronic Kidney Disease: Mechanism and Clinical Implications - A Narrative Review. *Int. J. Mol. Sci*. 2022;23:6047.
14. Post A, Tsikas D, Bakker SJL. Creatine is a Conditionally Essential Nutrient in Chronic Kidney Disease: A Hypothesis and Narrative Literature Review. *Nutrients*. 2019;11:1044.
15. Low S, Pek S, Moh A, Khin CYA, Lim CL, Ang SF, et al. Low muscle mass is associated with progression of chronic kidney disease and albuminuria - An 8-year longitudinal study in Asians with Type 2 Diabetes. *Diabetes Res Clin Pract*. 2021;174:108777.
16. Kim TN, Lee EJ, Hong JW, Kim JM, Won JC, Kim MK, et al. Relationship Between Sarcopenia and Albuminuria: The 2011 Korea National Health and Nutrition Examination Survey. *Medicine (Baltimore)*. 2016;95(3):e2500.
17. Cho JM, Koh JH, Kim SG, Lee S, Kim Y, Cho S, et al. Associations of MRI-derived kidney volume, kidney function, body composition and physical performance in ≈38 000 UK Biobank participants: a population-based observational study. *Clin Kidney J.* 2024;17(4):sfae068.
18. Tanaka M, Okada H, Hashimoto Y, Kumagai M, Yamaoka M, Nishimura H, et al. Trunk muscle quality and quantity are associated with renal volume in nondiabetic people. *Clin Kidney J.* 2023;16(12): 2597–2604.
19. Hamrick MW. The skeletal muscle secretome: an emerging player in muscle-bone crosstalk. *Bonekey Rep*. 2012;1:60.
20. Rondon-Berrios H, Wang Y, Mitch WE. Can muscle-kidney crosstalk slow progression of CKD? *J Am Soc Nephrol*. 2014;25:2681–3.
21. Hanatani S, Izumiya Y, Araki S, Rokutanda T, Kimura Y, Walsh K, et al. Akt1-mediated fast/glycolytic skeletal muscle growth attenuates renal damage in experimental kidney disease. *J Am Soc Nephrol*. 2014;25:2800–11.
22. Peng H, Wang Q, Lou T, Qin J, Jung S, Shetty V, et al. Myokine mediated muscle-kidney crosstalk suppresses metabolic reprogramming and fibrosis in damaged kidneys. *Nat Commun*. 2017;8:1493.
23. Severinsen MCK, Pedersen BK. Muscle-organ crosstalk: the emerging roles of myokines. *Endocr Rev*. 2020;41:594–609.
24. Solagna F, Tezze C, Lindenmeyer MT, Lu S, Wu G, Liu S, et al. Pro-cachectic factors link experimental and human chronic kidney disease to skeletal muscle wasting programs. *J Clin Invest*. 2021;131:e135821.
25. Peart WS. The kidney as an endocrine organ. *Lancet*. 1977;2:543–8.
26. Agapova OA, Fang Y, Sugatani T, Seifert ME, Hruska KA. Ligand trap for the activin type IIA receptor protects against vascular disease and renal fibrosis in mice with chronic kidney disease. *Kidney Int*. 2016;89:1231–43.
27. Thomas R, Kanso A, Sedor JR. Chronic kidney disease and its complications. *Prim Care.* 2008;35:329–44 vii.
28. Doi K, Ishizu T, Fujita T, Noiri E. Lung injury following acute kidney injury: kidney-lung crosstalk. *Clin Exp Nephrol.* 2011;15:464–70.
29. Singer MA. Of mice and men and elephants: metabolic rate sets glomerular filtration rate. *Am J Kidney Dis.* 2001;37(1):164-178.
30. Pigneur F, Luciani A, Ghosn M, Reizine E, Morel A, Stehlé T. Skeletal Muscle Density Is Highly Dependent on CT Instrumentation. *Radiology*. 2023;307(5):e222839.

**Annex III: Supplementary Tables**

Supplemental Table S1. Univariable linear regression analyses with mGFR

| mGFR (ml/min) | Total population | | | Age ≤53 years | | | Age >53 years | | |
| --- | --- | --- | --- | --- | --- | --- | --- | --- | --- |
|  | B (95% CI) | Std. β | *p* | B (95% CI) | Std. β | *p* | B (95% CI) | Std. β | *p* |
| SMA, cm^2^ | 0.35 (0.31; 0.38) | 0.57 | <0.001 | 0.30 (0.26; 0.35) | 0.53 | <0.001 | 0.33 (0.29; 0.37) | 0.59 | <0.001 |
| SMI, cm^2^/m^2^ | 0.46 (0.34; 0.58) | 0.25 | <0.001 | 0.29 (0.13; 0.45) | 0.17 | <0.001 | 0.46 (0.31; 0.61) | 0.27 | <0.001 |
| SMRA, HU | 0.58 (0.45; 0.71) | 0.28 | <0.001 | 0.29 (0.09; 0.49) | 0.13 | 0.01 | 0.40 (0.23; 0.57) | 0.21 | <0.001 |
| 24-h CER, mmol/24 hours | 0.67 (0.45; 0.89) | 0.20 | <0.001 | 0.32 (0.02; 0.61) | 0.10 | 0.04 | 0.46 (0.16; 0.76) | 0.15 | 0.002 |
| CER-index, mmol/24 hours/m | 1.31 (0.89; 1.73) | 0.21 | <0.001 | 0.66 (0.10; 1.22) | 0.11 | 0.02 | 0.91 (0.34; 1.47) | 0.15 | 0.002 |
| Female sex | -3.63 (-5.66; -1.60) | -0.12 | <0.001 | -2.00 (-4.69; 0.70) | -0.07 | 0.15 | -4.97 (-7.54; -2.41) | -0.18 | <0.001 |
| SBP, mmHg | -0.11 (-0.19; -0.03) | -0.09 | 0.01 | 0.04 (-0.08; 0.16) | 0.03 | 0.53 | -0.02 (-0.11; 0.08) | -0.02 | 0.75 |
| Age, years | -0.65 (-0.74; -0.57) | -0.45 | <0.001 | -0.40 (-0.58; -0.21) | -0.20 | <0.001 | -0.71 (-0.93; -0.49) | -0.29 | <0.001 |

^Body surface area, BSA (m2); measured glomerular filtration rate, mGFR (mL/min); skeletal muscle index, SMI (cm2/m2); skeletal muscle radiation attenuation, SMRA (Hounsfield units, HU); systolic blood pressure, SBP (mmHg); 24 hours urinary creatinine excretion rate, 24-h CER (mmol/24 hours); urinary creatinine excretion rate index, CER-index (24-h CER/height in meters, mmol/24 hours/m).^

Supplemental Table S2. Linear regression analyses with 24-UCE, per sex and age category

| mGFR (ml/min) | Age ≤53 years | | | | Age >53 years | | | |
| --- | --- | --- | --- | --- | --- | --- | --- | --- |
|  | B (95% CI) | Std. β | *p* | Adj. R^2^ | B (95% CI) | Std. β | *p* | Adj. R^2^ |
| **Female donors**  24-h CER  24-h CER  BSA  24-h CER  BSA  Age | 1.18 (0.60; 1.76)  0.54 (-0.03; 1.12)  45.8 (30.8; 60.9)  0.60 (0.05;1.14)  41.9 (27.4; 56.3)  -0.76 (-1.09; -0.44) | 0.27  0.12  0.40  0.14  0.36  -0.28 | <0.001  0.07  <0.001  0.03  <0.001  <0.001 | 0.07  0.20  0.28 | 0.32 (-0.28; 0.93)  0.07 (-0.53; 0.67)  26.5 (13.1; 39.9)  -0.02 (-0.58; 0.55)  22.4 (9.66; 35.1)  -0.81 (-1.12; -0.50) | 0.07  0.02  0.26  -0.004  0.22  -0.32 | 0.29  0.82  <0.001  0.95  <0.001  <0.001 | 0.001  0.06  0.16 |
| **Male donors**  24-h CER  24-h CER  BSA  24-h CER  BSA  Age | 1.06 (0.40; 1.72)  0.45 (-0.20; 1.09)  49.2 (32.5; 65.8)  0.45 (-0.19; 1.10)  50.0 (33.4; 66.6)  -0.27 (-0.57; 0.03) | 0.22  0.09  0.39  0.09  0.40  -0.11 | 0.002  0.17  <0.001  0.16  <0.001  0.08 | 0.04  0.18  0.19 | 1.63 (1.02; 2.24)  0.91 (0.23; 1.58)  35.6 (18.8; 52.4)  0.67 (0.01; 1.34)  32.9 (16.6; 49.2)  -0.79 (-1.18; -0.40) | 0.34  0.19  0.30  0.14  0.28  -0.25 | <0.001  0.01  <0.001  0.047  <0.001  <0.001 | 0.11  0.18  0.23 |

^Body surface area, BSA (m2); measured glomerular filtration rate, mGFR (mL/min); 24 hours urinary creatinine excretion rate, 24-h CER (mmol/24 hours).^

Supplemental Table S3. Multivariable linear regression analyses with mGFR standardized for BSA, per age category

| mGFR (ml/min per 1.73m^2^) | Age ≤53 years | | | | Age >53 years | | | |
| --- | --- | --- | --- | --- | --- | --- | --- | --- |
|  | B (95% CI) | Std. β | *p* | Adj. R^2^ | B (95% CI) | Std. β | *p* | Adj. R^2^ |
| SMA  Female sex | 0.07 (0.004; 0.14)  1.91 (-2.66; 6.47) | 0.17  0.07 | 0.04  0.41 | 0.01 | 0.10 (0.03; 0.17)  0.80 (-3.94; 5.52) | 0.24  0.03 | 0.01  0.74 | 0.04 |
| SMI  Female sex | 0.36 (0.15; 0.57)  1.88 (-1.62; 5.38) | 0.21  0.07 | <0.001  0.29 | 0.03 | 0.50 (0.28; 0.71)  0.88 (-2.70; 4.46) | 0.29  0.03 | <0.001  0.63 | 0.07 |
| SMRA  Female sex | 0.27 (0.07; 0.47)  -1.57 (-4.27; 1.13) | 0.13  -0.06 | 0.01  0.25 | 0.02 | 0.36 (0.19; 0.53)  -4.03 (-6.60; -1.47) | 0.19  -0.14 | <0.001  0.002 | 0.06 |
| 24-h CER  Female sex | 0.27 (-0.08; 0.62)  -0.83 (-4.16; 2.49) | 0.09  -0.03 | 0.14  0.62 | 0.01 | 0.19 (-0.18; 0.56)  -3.94 (-7.18; -0.70) | 0.06  -0.14 | 0.32  0.02 | 0.03 |
| CER-index  Female sex | 0.58 (-0.05; 1.22)  -0.88 (-4.02; 2.26) | 0.10  -0.03 | 0.07  0.58 | 0.01 | 0.46 (-0.21; 1.13)  -3.80 (-6.86; -0.74) | 0.08  -0.14 | 0.18  0.02 | 0.03 |
| SMA  Female sex  Age | 0.06 (-0.01; 0.12)  0.25 (-2.04; 6.96)  -0.37 (-0.56; -0.18) | 0.14  0.09  -0.19 | 0.08  0.28  <0.001 | 0.04 | 0.05 (-0.02; 0.12)  -1.78 (-6.43; 2.88)  -0.66 (-0.88; -0.44) | 0.13  -0.06  -0.26 | 0.14  0.45  <0.001 | 0.11 |
| SMI  Female sex  Age | 0.35 (0.15; 0.56)  3.00 (-0.48; 6.48)  -0.38 (-0.56; -0.20) | 0.21  0.11  -0.19 | <0.001  0.09  <0.001 | 0.06 | 0.41 (0.20; 0.62)  0.03 (-3.44; 3.50)  -0.64 (-0.86; -0.42) | 0.24  0.001  -0.26 | <0.001  0.99  <0.001 | 0.13 |
| SMRA  Female sex  Age | 0.17 (-0.04; 0.38)  -0.69 (-3.40; 2.03)  -0.33 (-0.53; -0.14) | 0.08  -0.02  -0.17 | 0.11  0.62  <0.001 | 0.04 | 0.27 (0.10; 0.44)  -4.06 (-6.54; -1.58)  -0.63 (-0.85; -0.41) | 0.14  -0.14  -0.25 | 0.002  0.001  <0.001 | 0.12 |
| 24-h CER  Female sex  Age | 0.28 (-0.07; 0.63)  0.34 (-2.96; 3.65)  -0.40 (-0.59; -0.21) | 0.09  0.01  -0.20 | 0.11  0.84  <0.001 | 0.05 | 0.00 (-0.36; 0.36)  -4.77 (-7.89; -1.64)  -0.68 (-0.90; -0.45) | 0.00  -0.17  -0.28 | 1.00  0.003  <0.001 | 0.10 |
| CER-index  Female sex  Age | 0.63 (0.01; 1.25)  0.36 (-2.77; 3.49)  -0.41 (-0.60; -0.22) | 0.11  0.01  -0.21 | 0.047  0.82  <0.001 | 0.05 | 0.16 (-0.49; 0.82)  -4.36 (-7.32; -1.41)  -0.67 (-0.89; -0.45) | 0.03  -0.16  -0.27 | 0.62  0.004  <0.001 | 0.10 |

^Body surface area, BSA (m2); measured glomerular filtration rate, mGFR (mL/min); skeletal muscle index, SMI (cm2/m2); skeletal muscle radiation attenuation, SMRA (Hounsfield units, HU); 24 hours urinary creatinine excretion rate, 24-h CER (mmol/24 hours); urinary creatinine excretion rate index, CER-index (24-h CER/height in meters, mmol/24 hours/m).^

Supplemental Table S4. Univariable linear regression analyses with skeletal muscle parameters

|  | Total population | | | Age ≤53 years | | | Age >53 years | | |
| --- | --- | --- | --- | --- | --- | --- | --- | --- | --- |
| **SMA** | B (95% CI) | Std. β | *p* | B (95% CI) | Std. β | *p* | B (95% CI) | Std. β | *p* |
| mGFR, mL/min | 0.94 (0.85; 1.02) | 0.57 | <0.001 | 0.93 (0.79; 1.07) | 0.53 | <0.001 | 1.04 (0.91; 1.18) | 0.59 | <0.001 |
| Female sex | -56.1 (-58.7; -53.5) | -0.81 | <0.001 | -56.2 (-60.0; -52.4) | -0.81 | <0.001 | -55.8 (-59.0; -52.5) | -0.84 | <0.001 |
| SBP, mmHg | 0.51 (0.34; 0.68) | 0.19 | <0.001 | 0.85 (0.57; 1.13) | 0.27 | <0.001 | 0.52 (0.31; 0.73) | 0.22 | <0.001 |
| Age, years | -0.78 (-0.98; -0.58) | -0.25 | <0.001 | -1.01 (-1.45; -0.58) | -0.21 | <0.001 | -0.96 (-1.48; -0.44) | -0.16 | <0.001 |
| BSA, m^2^ | 132.0 (124.3; 140.0) | 0.74 | <0.001 | 132.6 (121.3; 144.0) | 0.74 | <0.001 | 128.0 (117.5; 138.6) | 0.74 | <0.001 |
| BSA-standardized mGFR, mL/min per 1.73m^2^ | 0.48 (0.33; 0.62) | 0.21 | <0.001 | 0.28 (0.05; 0.51) | 0.12 | 0.02 | 0.52 (0.31; 0.74) | 0.22 | <0.001 |
| **SMI** |  |  |  |  |  |  |  |  |  |
| mGFR, mL/min | 0.18 (0.16; 0.21) | 0.46 | <0.001 | 0.17 (0.14; 0.21) | 0.41 | <0.001 | 0.22 (0.18; 0.25) | 0.49 | <0.001 |
| Female sex | -11.3 (-12.1; -10.5) | -0.68 | <0.001 | -10.8 (-12.0; -9.59) | -0.65 | <0.001 | -11.8 (-12.9; -10.7) | -0.71 | <0.001 |
| SBP, mmHg | 0.12 (0.08; 0.16) | 0.18 | <0.001 | 0.18 (0.11; 0.25) | 0.24 | <0.001 | 0.12 (0.07; 0.18) | 0.21 | <0.001 |
| Age, years | -0.14 (-0.19; -0.09) | -0.18 | <0.001 | -0.17 (-0.28; -0.06) | -0.15 | 0.002 | -0.18 (-0.31; -0.04) | -0.12 | 0.01 |
| BSA, m^2^ | 21.1 (18.6; 23.6) | 0.49 | <0.001 | 20.4 (16.8; 23.9) | 0.47 | <0.001 | 21.2 (17.7; 24.8) | 0.48 | <0.001 |
| BSA-standardized mGFR, mL/min per 1.73m^2^ | 0.13 (0.10; 0.17) | 0.25 | <0.001 | 0.10 (0.04; 0.15) | 0.17 | <0.001 | 0.16 (0.11; 0.21) | 0.27 | <0.001 |
| **SMRA** |  |  |  |  |  |  |  |  |  |
| mGFR, mL/min | 0.04 (0.01; 0.06) | 0.11 | 0.002 | -0.02 (-0.06; 0.01) | -0.07 | 0.14 | 0.01 (-0.03; 0.05) | 0.03 | 0.56 |
| Female sex | -1.90 (-2.89; -0.91) | -0.13 | <0.001 | -1.39 (-2.66; -0.13) | -0.10 | 0.03 | -2.31 (-3.69; -0.94) | -0.15 | 0.001 |
| SBP, mmHg | -0.10 (-0.13; -0.06) | -0.16 | <0.001 | -0.05 (-0.11; 0.003) | -0.09 | 0.06 | -0.05 (-0.10; -0.004) | -0.10 | 0.03 |
| Age, years | -0.27 (-0.32; -0.23) | -0.40 | <0.001 | -0.28 (-0.36; -0.20) | -0.30 | <0.001 | -0.26 (-0.38; -0.14) | -0.20 | <0.001 |
| BSA, m^2^ | -9.42 (-11.9; -6.91) | -0.24 | <0.001 | -10.1 (-13.3; -6.97) | -0.29 | <0.001 | -11.2 (-14.8; -7.73) | -0.28 | <0.001 |
| BSA-standardized mGFR, mL/min per 1.73m^2^ | 0.14 (0.11; 0.17) | 0.28 | <0.001 | 0.06 (0.02; 0.11) | 0.13 | 0.01 | 0.11 (0.07; 0.16) | 0.21 | <0.001 |
| **24-h CER** |  |  |  |  |  |  |  |  |  |
| mGFR, mL/min | 0.11 (0.09; 0.12) | 0.48 | <0.001 | 0.10 (0.08; 0.12) | 0.42 | <0.001 | 0.11 (0.09; 0.13) | 0.47 | <0.001 |
| Female sex | -5.22 (-5.74; -4.70) | -0.56 | <0.001 | -5.16 (-5.92; -4.39) | -0.55 | <0.001 | -5.27 (-5.93; -4.61) | -0.61 | <0.001 |
| SBP, mmHg | 0.05 (0.03; 0.08 | 0.15 | <0.001 | 0.11 (0.07; 0.15) | 0.25 | <0.001 | 0.06 (0.03; 0.09) | 0.18 | <0.001 |
| Age, years | -0.11 (-0.13; -0.08) | -0.24 | <0.001 | -0.06 (-0.12; 0.004) | -0.09 | 0.07 | -0.11 (-0.19; -0.04) | -0.15 | 0.002 |
| BSA, m^2^ | 14.3 (13.0; 15.6) | 0.59 | <0.001 | 13.8 (11.8; 15.7) | 0.56 | <0.001 | 14.1 (12.4; 15.8) | 0.62 | <0.001 |
| BSA-standardized mGFR, mL/min per 1.73m^2^ | 0.06 (0.04; 0.08) | 0.20 | <0.001 | 0.03 (0.002; 0.07) | 0.10 | 0.04 | 0.05 (0.02; 0.08) | 0.15 | 0.002 |

^Body surface area, BSA (m2); measured glomerular filtration rate, mGFR (mL/min); skeletal muscle index, SMI (cm2/m2); skeletal muscle radiation attenuation, SMRA (Hounsfield units, HU); systolic blood pressure, SBP (mmHg); 24 hours urinary creatinine excretion rate, 24-h CER (mmol/24 hours).^

Supplemental Table S5. Multivariable linear regression analyses with skeletal muscle parameters, per age category

|  | Age ≤53 years | | | | Age >53 years | | | |
| --- | --- | --- | --- | --- | --- | --- | --- | --- |
|  | B (95% CI) | Std. β | *p* | Adj. R^2^ | B (95% CI) | Std. β | *p* | Adj. R^2^ |
| **SMA** |  |  |  |  |  |  |  |  |
| mGFR  Female sex | 0.40 (0.31; 0.50)  -50.0 (-53.9; -46.1) | 0.23  -0.72 | <0.001  <0.001 | 0.72 | 0.40 (0.30; 0.49)  -49.2 (-52.8; -45.6) | 0.22  -0.73 | <0.001  <0.001 | 0.75 |
| mGFR  Female sex  SBP | 0.39 (0.29; 0.49)  -49.2 (-53.1; -45.3)  0.18 (0.02; 0.34) | 0.23  -0.71  0.06 | <0.001  <0.001  0.03 | 0.72 | 0.40 (0.31; 0.50)  -48.9 (-52.6; -45.2)  0.05 (-0.07; 0.16) | 0.23  -0.73  0.02 | <0.001  <0.001  0.43 | 0.75 |
| mGFR  Female sex  SBP  BSA | 0.20 (0.10; 0.29)  -39.6 (-43.7; -35.5)  0.07 (-0.08; 0.22)  56.1 (44.4; 67.9) | 0.11  -0.57  0.02  0.31 | <0.001  <0.001  0.34  <0.001 | 0.77 | 0.25 (0.16; 0.34)  -39.4 (-43.3; -35.6)  0.03 (-0.08; 0.13)  50.9 (40.4; 61.3) | 0.14  -0.59  0.01  0.29 | <0.001  <0.001  0.64  <0.001 | 0.79 |
| mGFR  Female sex  SBP  BSA  Age | 0.18 (0.08; 0.28)  -39.1 (-43.2; -34.9)  0.09 (-0.06; 0.24)  56.5 (44.8; 68.3)  -0.19 (-0.42; 0.04) | 0.10  -0.56  0.03  0.32  -0.04 | <0.001  <0.001  0.23  <0.001  0.10 | 0.77 | 0.21 (0.12; 0.31)  -40.2 (-44.0; -36.3)  0.04 (-0.07; 0.15)  50.1 (39.7; 60.4)  -0.36 (-0.62; -0.09) | 0.12  -0.60  0.02  0.28  -0.06 | <0.001  <0.001  0.45  <0.001  0.01 | 0.79 |
| **SMI** |  |  |  |  |  |  |  |  |
| mGFR  Female sex | 0.07 (0.04; -0.10)  -9.71 (-11.0; -8.45) | 0.17  -0.59 | <0.001  <0.001 | 0.46 | 0.08 (0.05; 0.11)  -10.3 (-11.5; -9.10) | 0.18  -0.62 | <0.001  <0.001 | 0.53 |
| mGFR  Female sex  SBP | 0.07 (0.04; 0.10)  -9.50 (-10.8; -8.22)  0.05 (-0.01; 0.10) | 0.16  -0.58  0.07 | <0.001  <0.001  0.08 | 0.46 | 0.08 (0.05; 0.11)  -10.1 (-11.3; -8.87)  0.03 (-0.01; 0.07) | 0.19  -0.61  0.05 | <0.001  <0.001  0.13 | 0.53 |
| mGFR  Female sex  SBP  BSA | 0.07 (0.03; 0.10)  -9.50 (-11.0; -8.02)  0.05 (-0.01; 0.10)  0.003 (-4.23; 4.23) | 0.16  -0.58  0.07  0.00 | <0.001  <0.001  0.08  1.00 | 0.46 | 0.09 (0.05; 0.12)  -10.3 (-11.8; -8.91)  0.03 (-0.01; 0.07)  -1.29 (-5.13; 2.55) | 0.20  -0.62  0.05  -0.03 | <0.001  <0.001  0.12  0.51 | 0.53 |
| mGFR  Female sex  SBP  BSA  Age | 0.07 (0.03; 0.10)  -9.49 (-11.0; -8.00)  0.05 (-0.01; 0.10)  0.01 (-4.23; 4.25)  -0.003 (-0.09; 0.08) | 0.16  -0.58  0.07  0.00  -0.002 | <0.001  <0.001  0.08  1.00  0.95 | 0.46 | 0.08 (0.04; 0.11)  -10.5 (-12.0; -9.08)  0.03 (-0.01; 0.07)  -1.48 (-5.32; 2.35)  -0.09 (-0.19; 0.01) | 0.17  -0.63  0.06  -0.03  -0.06 | <0.001  <0.001  0.09  0.45  0.09 | 0.53 |
| **SMRA** |  |  |  |  |  |  |  |  |
| mGFR  Female sex | -0.05 (-0.08; -0.01)  -2.23 (-3.62; -0.83) | -0.14  -0.17 | 0.01  0.002 | 0.02 | -0.03 (-0.07; 0.02)  -2.79 (-4.37; -1.21) | -0.06  -0.19 | 0.23  <0.001 | 0.02 |
| mGFR  Female sex  SBP | -0.04 (-0.08; -0.01)  -2.53 (-3.94; -1.12)  -0.07 (-0.13; -0.01) | -0.13  -0.19  -0.11 | 0.01  <0.001  0.03 | 0.03 | -0.03 (-0.07; 0.01)  -3.34 (-4.94; -1.74)  -0.08 (-0.13; -0.03) | -0.07  -0.22  -0.15 | 0.18  <0.001  0.002 | 0.04 |
| mGFR  Female sex  SBP  BSA | 0.03 (-0.004; 0.07)  -6.32 (-7.78; -4.86)  -0.03 (-0.08; 0.03)  -21.7 (-25.9; -17.6) | 0.09  -0.47  -0.05  -0.63 | 0.08  <0.001  0.32  <0.001 | 0.22 | 0.05 (0.02; 0.09)  -8.50 (-10.1; -6.91)  -0.07 (-0.11; -0.02)  -27.7 (-32.0; -23.5) | 0.14  -0.57  -0.12  -0.70 | 0.01  <0.001  0.003  <0.001 | 0.30 |
| mGFR  Female sex  SBP  BSA  Age | 0.01 (-0.03; 0.04)  -5.53 (-6.94; -4.13)  0.001 (-0.05; 0.05)  -21.1 (-25.1; -17.1)  -0.27 (-0.35; -0.20) | 0.02  -0.41  0.002  -0.61  -0.30 | 0.64  <0.001  0.96  <0.001  <0.001 | 0.30 | 0.02 (-0.02; 0.05)  -9.19 (-10.7; -7.66)  -0.05 (-0.09; -0.01)  -28.5 (-32.6; -24.4)  -0.34 (-0.45; -0.24) | 0.04  -0.61  -0.10  -0.72  -0.26 | 0.41  <0.001  0.02  <0.001  <0.001 | 0.35 |
| **24-h CER** |  |  |  |  |  |  |  |  |
| mGFR  Female sex | 0.05 (0.03; 0.07)  -4.30 (-5.12; -3.48) | 0.23  -0.46 | <0.001  <0.001 | 0.34 | 0.05 (0.03; 0.07)  -4.32 (-5.06; -3.58) | 0.22  -0.50 | <0.001  <0.001 | 0.40 |
| mGFR  Female sex  SBP | 0.05 (0.03; 0.07)  -4.11 (-4.94; -3.28)  0.04 (0.01; 0.08) | 0.21  -0.44  0.10 | <0.001  <0.001  0.02 | 0.35 | 0.05 (0.03; 0.07)  -4.23 (-4.99; -3.47)  0.01 (-0.01; 0.04) | 0.22  -0.49  0.04 | <0.001  <0.001  0.29 | 0.40 |
| mGFR  Female sex  SBP  BSA | 0.03 (0.004; 0.05)  -2.94 (-3.86; -2.02)  0.03 (-0.01; 0.06)  6.97 (4.30; 9.63) | 0.11  -0.31  0.07  0.29 | 0.02  <0.001  0.11  <0.001 | 0.39 | 0.03 (0.01; 0.05)  -2.76 (-3.58; -1.93)  0.01 (-0.01; 0.03)  7.91 (5.71; 10.1) | 0.11  -0.32  0.04  0.35 | 0.01  <0.001  0.34  <0.001 | 0.46 |
| mGFR  Female sex  SBP  BSA  Age | 0.03 (0.01; 0.05)  -2.98 (-3.91; -2.05)  0.03 (-0.01; 0.06)  6.93 (4.26; 9.60)  0.02 (-0.04; 0.07) | 0.12  -0.32  0.06  0.28  0.03 | 0.02  <0.001  0.13  <0.001  0.54 | 0.39 | 0.02 (-0.001; 0.04)  -2.88 (-3.71; -2.04)  0.01 (-0.01; 0.04)  7.79 (5.59; 9.99)  -0.06 (-0.11; 0.002) | 0.09  -0.33  0.04  0.34  -0.07 | 0.07  <0.001  0.25  <0.001  0.06 | 0.46 |
| **CER-index** |  |  |  |  |  |  |  |  |
| mGFR  Female sex | 0.03 (0.01; 0.04)  -1.89 (-2.35; -1.43) | 0.20  -0.38 | <0.001  <0.001 | 0.25 | 0.02 (0.01; 0.04)  -2.03 (-2.44; -1.61) | 0.19  -0.44 | <0.001  <0.001 | 0.32 |
| mGFR  Female sex  SBP | 0.02 (0.01; 0.04)  -1.80 (-2.27; -1.34)  0.02 (0.003; 0.04) | 0.19  -0.37  0.10 | <0.001  <0.001  0.02 | 0.26 | 0.02 (0.01; 0.04)  -1.97 (-2.40; -1.55)  0.01 (-0.01; 0.02) | 0.20  -0.43  0.05 | <0.001  <0.001  0.23 | 0.32 |
| mGFR  Female sex  SBP  BSA | 0.02 (0.003; 0.03)  -1.40 (-1.93; -0.87)  0.02 (-0.001; 0.04)  2.41 (0.88; 3.93) | 0.12  -0.28  0.08  0.19 | 0.02  <0.001  0.07  0.002 | 0.28 | 0.02 (0.003; 0.03)  -1.41 (-1.88; -0.93)  0.01 (-0.01; 0.02)  3.04 (1.77; 4.31) | 0.12  -0.31  0.05  0.25 | 0.01  <0.001  0.26  <0.001 | 0.35 |
| mGFR  Female sex  SBP  BSA  Age | 0.02 (0.004; 0.03)  -1.44 (-1.97; -0.90)  0.02 (-0.003; 0.04)  2.37 (0.84; 3.90)  0.02 (-0.02; 0.04) | 0.13  -0.29  0.07  0.18  0.04 | 0.01  <0.001  0.10  0.002  0.33 | 0.28 | 0.01 (-0.001; 0.02)  -1.47 (-1.95; -0.99)  0.01 (-0.004; 0.02)  2.98 (1.71; 4.24)  -0.03 (-0.06; 0.004) | 0.09  -0.32  0.05  0.25  -0.07 | 0.07  <0.001  0.19  <0.001  0.08 | 0.35 |

^Body surface area, BSA (m2); measured glomerular filtration rate, mGFR (mL/min); skeletal muscle index, SMI (cm2/m2); skeletal muscle radiation attenuation, SMRA (Hounsfield units, HU); systolic blood pressure, SBP (mmHg); 24 hours urinary creatinine excretion rate, 24-h CER (mmol/24 hours); urinary creatinine excretion rate index, CER-index (24-h CER/height in meters, mmol/24 hours/m).^

**Annex IV: Supplementary Figures**


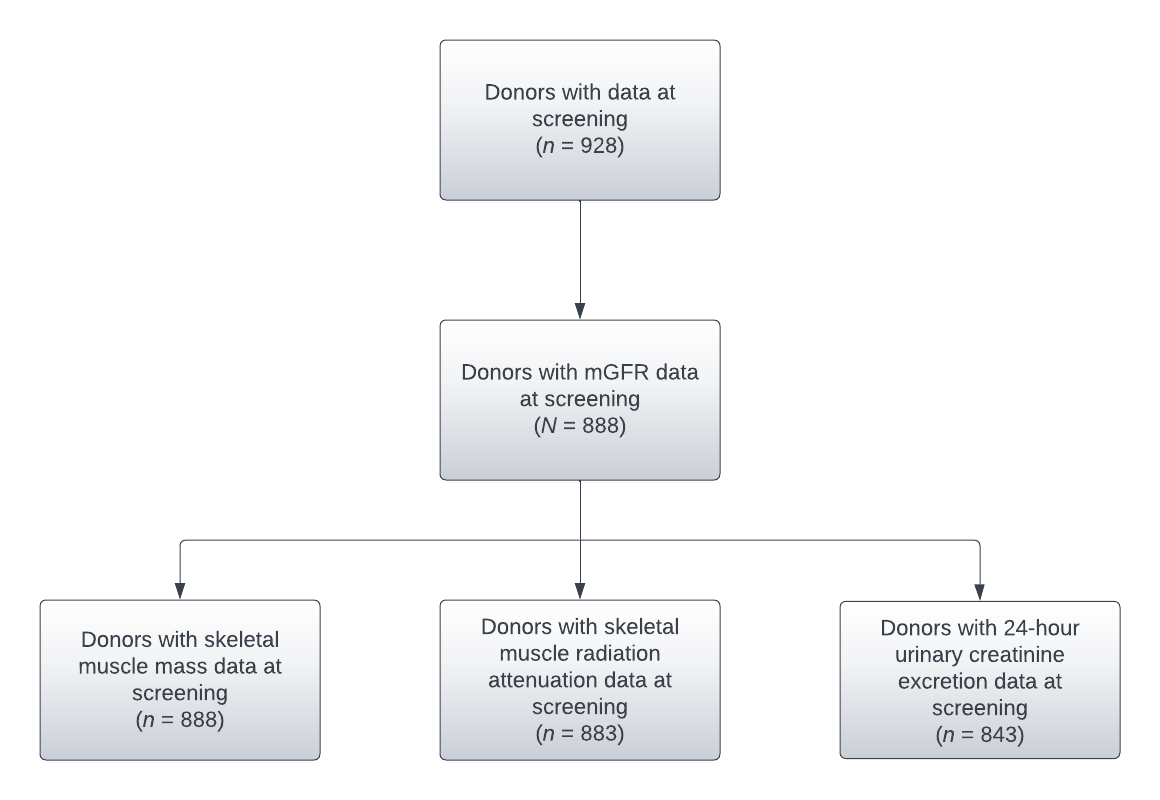


Supplemental Figure S1. Overview of the study participants.

^Measured glomerular filtration rate, mGFR.^


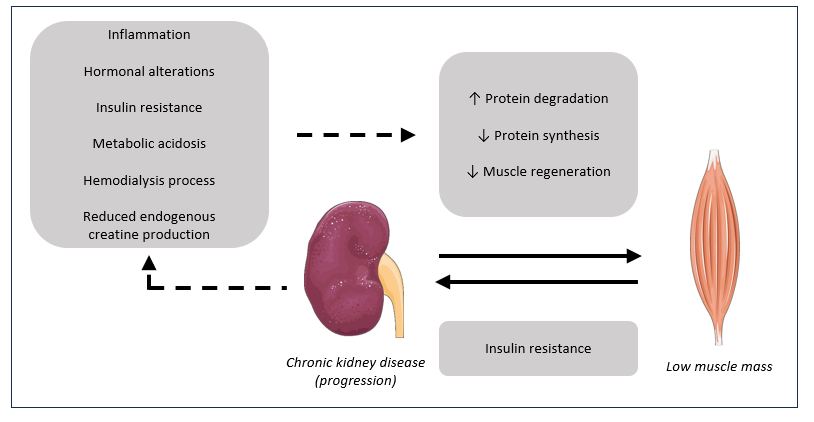


Supplemental Figure S2. Illustration of the in literature described mechanisms underlying the relationship between muscle and kidney in patients with chronic kidney disease. The graphics depicted in this illustration were adapted from Servier Medical Art (https://smart.servier.com/).
